# Logical Connectives Modulate Attention to Simulations Evoked by the Constituents They Link Together

**DOI:** 10.3389/fpsyg.2018.01358

**Published:** 2018-08-03

**Authors:** Magda L. Dumitru, Gitte H. Joergensen

**Affiliations:** ^1^Psychology and Neuroscience of Cognition Research Unit, University of Liège, Liège, Belgium; ^2^School of Psychology, University of Connecticut, Mansfield, CT, United States; ^3^Department of Psychology, University of York, York, United Kingdom

**Keywords:** logical connective, visual attention, language simulation, language Gestalt, mental imagery

## Abstract

In previous studies investigating logical-connectives simulations, participants focused their attention on verifying truth-condition satisfaction for connective expressions describing visual stimuli (e.g., Dumitru, 2014; Dumitru and Joergensen, 2016). Here, we sought to replicate and extend the findings that conjunction and disjunction simulations are structured as one and two Gestalts, respectively, by using language – picture matching tasks where participants focused their attention exclusively on stimuli visuospatial properties. Three studies evaluated perceptual compatibility effects between visual displays varying stimuli direction, size, and orientation, and basic sentences featuring the logical connectives AND, OR, BUT, IF, ALTHOUGH, BECAUSE, and THEREFORE (e.g., “There is blue AND there is red”). Response times highlight correlations between the Gestalt arity of connective simulations and visual attention patterns, such that words referring to constituents in the same Gestalt were matched faster to visual stimuli displayed sequentially rather than alternatively, having the same size rather than different sizes, and being oriented along axes other than horizontal. The results also highlight attentional patterns orthogonal to Gestalt arity: visual stimuli corresponding to simulation constituents were processed faster when they appeared onscreen from left to right than from right to left, when they were emphasized or de-emphasized together (i.e., faster processing of all-small or all-large stimuli pairs), and when they formed a downward-oriented diagonal, which signals a simulation boundary. More generally, our findings suggest that logical connectives rapidly evoke simulations that trigger top-down attention patterns over the grouping and properties of visual stimuli corresponding to the constituents they link together.

## Introduction

Language commands an impressive battery of devices for encoding objects and events in the world. Although “The shark eats the fish” and “The fish is eaten by the shark” both capture the same event, they draw attention, respectively, to the agent setting the event in motion and to the patient undergoing the action (Tomlin, 1997). Similarly, speakers designate larger, steadier, or more important items as anchors for smaller, less steady, or less important items when uttering “The bike is near the house"” rather than “The house is near the bike” (Talmy, 2000). The former sentence suggests that ‘the bike’ is the item most likely to undergo change and thus most deserving of attention, hence it receives the most prominent role in the sentence (i.e., subject). What ultimately drives the choice of language structures are the simulations of concrete objects and events that words and sentences instantly evoke (Glenberg, 1997; Zwaan and Radvansky, 1998; Barsalou, 1999; MacWhinney, 1999; Glenberg and Kaschak, 2002; Zwaan, 2004; Zwaan and Taylor, 2006; Barsalou et al., 2008). When talking about complex events, individuals re-enact visual scenes as they imagine or remember them and, in the process, shift their gaze between visual pointers (Ballard et al., 1997) or between spatial indices (Pylyshyn, 1989) even in the absence of visual stimuli (Spivey and Geng, 2001). In the present work, we argue that language does more than encode information on objects and events in the world via morphology or syntax, and more than evoke simulations of objects in virtual scenes (Johnson-Laird, 1983) that match visual objects. Language also encodes attention patterns that must be deployed over other language-evoked simulations that is, instructions on how to navigate visual scenes in the mind’s eye, which objects to group together, and which items within groups to emphasize. In particular, we aim to show that logical connectives modulate attention patterns over simulations evoked by the constituents they link together.

A tacit assumption in studies inspired by theories of embodied and grounded cognition has been that language-evoked simulations are updated by combining new and already acquired information in componential fashion. Specifically, the meaning of the whole is expected to be retrieved from the meaning of the parts, and the other way around. In contrast, recent evidence points to a novel dimension of simulation formation, as language comprehenders were shown to instantly package information in chunks that display Gestalt-like properties (Dumitru et al., 2013; Dumitru and Taylor, 2014; Dumitru, 2016; Dumitru and Joergensen, 2016). Gestalts depart from the common notion of ‘chunks’ in several ways. First, the simulations evoked by their components are likely to be fused together, rather than remain independent. As originally described, Gestalts are units of visual information that are built following the principle of the whole being “different from the sum of its parts” (Koffka, 1922; Wertheimer, 1923; Koehler, 1929), with consequences for working memory processes and attention patterns. For example, a flock of birds moving together from left to right is perceived as a group, thereby forming one Gestalt according to the principle of ‘common fate.’ The characteristics of the whole Gestalt that is, the continuous motion of the group across the visual field, is the information most likely to be remembered about the birds in the group, rather than the properties of Gestalt parts that is, of the exemplars forming the flock.

Similarly, previous evidence detailed in the studies mentioned above points to the existence of “Gestalts of language.” We obtained higher accuracy scores for expressions such as “the purple AND the green” applied to visual displays containing two matching objects which appeared and disappeared on screen simultaneously rather than alternatively, were situated close to each other rather than far away from each other, and exemplified the same category (e.g., two disks) rather than different categories (e.g., one disk and one triangle). In other words, participants applied the Gestalt principles of common fate, proximity, and similarity to determine whether visual stimuli formed a single Gestalt, and if so, to accept descriptions by AND expressions. This suggests that the simulation evoked by the connective AND also comprises a single language Gestalt, which includes the simulations evoked by “purple” and by “green.” For the connective OR, the criteria were reversed such that participants validated disjunction expressions when two disks moved independently of each other, were placed far from each other, or were dissimilar, thereby suggesting that the simulation evoked by the connective OR includes two language Gestalts, evoked by “purple” and by “green.”

This evidence was elicited in reasoning tasks (Dumitru et al., 2013; Dumitru, 2014; Dumitru and Joergensen, 2016) as well as in memory tasks (Dumitru and Taylor, 2014) and opens the possibility that the simulations evoked by constituents of AND expressions are merged together in working memory and/or that cortical responses to connective expressions differ from responses elicited by individual components. These possibilities will be investigated in subsequent studies. In the current work, we explore the simulations evoked by a number of frequently occurring logical connectives (AND, OR, BUT, IF, ALTHOUGH, BECAUSE, and THEREFORE) in matching tasks between basic sentences (e.g., ‘There is purple AND there is orange’) and binary visual displays. We aim to show that connective simulations require deployment of specific attention patterns over the simulations evoked by the components they link together and that language sets in motion a hierarchy of word simulations by allowing lexical items such as logical connectives to provide instructions on how to modify the simulations evoked by co-occurring language items.

Another way in which Gestalts differ from simple chunks is that the former are embodied representations, hence are organized according to basic principles of perception and action. The number and structure of Gestalts built in working memory will depend on individuals’ experience with situations where they routinely use the connective expressions that evoke them. So, for example, since individuals typically use the connective AND (e.g., ‘coffee and biscuits’) in situations where both items linked by the connective are available, which results in joint selection, the representations of ‘coffee’ and ‘biscuits’ are fused into a single Gestalt. Therefore, we reason that attention should target them equally. Also, since individuals typically use the connective OR (e.g., ‘coffee or tea’) in situations where the items mentioned are not available at the same time or under the same conditions, which results in single selection, the representations of ‘coffee’ and ‘tea’ are kept in separate Gestalts. Therefore, we reason that attention should target the two items differently.

The third difference between chunks and Gestalts is that the latter are not ruled by the limitations specific to working-memory processes, which cover a span of approximately 5 units (Miller, 1956; Cowan, 2001). One may hypothesize that grouping word simulations into Gestalts might be a good strategy for reducing the number of items kept in working memory, thus improving cognitive performance. However, even though the conjunction expression ‘coffee and tea’ and the disjunction expression ‘coffee or tea’ each contain two nouns, they are organized in one Gestalt and in two Gestalts, respectively. Since there is no difference, from the point of view of working-memory limitations, in processing one or two units, the reason for organizing items into one or two Gestalts must reflect structural concerns and attention-based strategies. Building a specific number of Gestalts for specific connective expressions allows language users to subsequently highlight entire Gestalts or some of their components to facilitate later reference and streamline integration with prior or subsequent information in texts and utterances.

Unlike previous studies (e.g., Dumitru and Joergensen, 2016), where participants observed reasoning rules (i.e., they validated only trials where both visual stimuli matched the items mentioned in conjunction expressions and where at least one visual stimulus matched one of the items mentioned in disjunction expressions), the current studies aimed to determine whether connective expressions evoke Gestalt-based simulations irrespective of whether or not individuals engage in reasoning tasks, namely as soon as the connective is being mentioned. Importantly, by asking participants to decide on the match between two colors mentioned in connective sentences (e.g., “There is purple AND there is orange”) and the colors of two disks presented onscreen, we allowed for covert retrieval of connectives’ meaning. To facilitate the task, visual displays were either completely matching the colors mentioned (e.g., a purple disk next to an orange disk), or completely mismatching them (e.g., a blue disk next to a yellow disk). In other words, the matching tasks directed participants to use exclusively the information provided by the color names.

We investigated whether the connectives AND, OR, BUT, IF, ALTHOUGH, BECAUSE, and THEREFORE instantly evoke Gestalt-like simulations that modulate attention patterns over the simulations evoked by the components they link together. In particular, we presented participants with connective sentences (e.g., “There is blue AND there is red”) as well as with binary visual stimuli (i.e., two disks of different colors) for which we varied the dynamics (sequential or alternative presentation) and direction (left-to-right or right-to-left deployment), the size (equal or unequal, as well as increasing or decreasing stimuli), and the orientation (vertical, horizontal, diagonally rising, and diagonally falling placement). We predicted that, if a given connective provides instructions for grouping together word simulations in a single Gestalt, the corresponding visual stimuli would share certain properties (e.g., have the same size), hence the amount of attention allocated to each visual stimulus and thereby to each constituent simulation would be the same. In contrast, we predicted that, if a given connective provides instructions for grouping together word simulations in two Gestalts, the corresponding visual stimuli would have different properties (e.g., different sizes), hence the amount of attention allocated to each visual stimulus, and thereby to each constituent simulation, would vary. Moreover, we assumed that connective simulations mirror the characteristics of those visual displays for which participants are fastest to identify a match. Indeed, disjunction expressions are processed faster when constituent concepts are related (e.g., ‘doctor or nurse’) than when they are unrelated (e.g., ‘doctor or electrician’), suggesting that the two Gestalts evoked by disjunction are distinct exemplars of a semantic category (Dumitru and Taylor, 2014).

It has further been shown that words organize spatial relations along the basic dimensions ‘up’ and ‘down’ or ‘right’ and ‘left’ (e.g., Piaget, 1927; Zwaan and Yaxley, 2003; Meier and Robinson, 2004; Estes et al., 2008; Louwerse and Jeuniaux, 2010; Boroditsky et al., 2011; Dudschig et al., 2015). For example, ‘sun’ and ‘joy’ but also ‘key,’ ‘claw,’ and ‘baby’ were shown to evoke the ‘up’ direction, whereas ‘basement,’ ‘bleak,’ ‘milk,’ ‘pompous,’ and ‘Monday’ would evoke the ‘down’ direction (Goodhew and Kidd, 2016). More generally, individuals associate good things with ‘up’ and bad things with ‘down.’ Along the orthogonal axis, stimuli and actions perceived or performed with the right-side of the body were found to bear positive valence, whereas stimuli and actions perceived or performed with the left-side of the body would bear negative valence (Natale et al., 1983; Davidson, 1992). These tendencies are often cultural, but may also become part of language meaning and/or influence attention patterns. For instance, the overall preference for initial right hemisphere activation, which leads to a bias of attention to the left hemifield in spatial tasks such as drawing, visual scene processing, and numerical cognition, is modulated at least to a certain extent by cultural conventions favoring either left-to-right or right-to-left processing (Dehaene, 1992; Dehaene et al., 1993; Vaid, 1995, 1998; Chokron and De Agostini, 2000). Attention shifts from left to right during processing language or visual displays are further accounted for by the tendency towards approaching stimuli on the right side (Davidson et al., 1990; Schiff and Bassel, 1996), which is regulated by handedness (Casasanto, 2009). In our studies, we avoided broad approximations (e.g., ‘up’ or ‘down’), thus we fine-tuned the characterization of language simulations by also accommodating two diagonal orientations. We thereby targeted attention patterns that are relevant not only for determining Gestalt arity, but also for highlighting other types of attention biases.

Each of the three experiments we conducted included the seven connectives tested together in groups of two or three, in order to enhance the contrast between them and ensure that participants were able to covertly retrieve their meaning that is, the connective simulations. We tested together AND, OR, and BUT, then IF and ALTHOUGH, and finally BECAUSE and THEREFORE. Participants were assigned to one of three groups and completed the three studies while being tested on experimental conditions for each connective once. Specifically, the first group was presented with trials varying stimuli dynamics and direction for the connectives AND, OR, and BUT, with trials varying stimuli size for the connectives IF and ALTHOUGH, and with trials varying stimuli orientation for the connectives BECAUSE and THEREFORE. The second group was presented with trials varying stimuli dynamics and direction for the connectives IF and ALTHOUGH, with trials varying stimuli size for the connectives BECAUSE and THEREFORE, and with trials varying stimuli orientation for the connectives AND, OR, and BUT. The third group was presented with trials varying stimuli dynamics and direction for the connectives BECAUSE and THEREFORE, with trials varying stimuli size for the connectives AND, OR, and BUT, and with trials varying stimuli orientation for the connectives IF and ALTHOUGH. We analyzed response times for each connective and for each experiment separately, hence data reported in each analysis were obtained within subjects.

All volunteers were students at the University of York, received course credit for their participation, and signed an informed consent form upon enrolment in the study, in accordance with the Declaration of Helsinki. The protocol was approved by the Ethics Committee of the University of York. For each study, and for each group of connective expressions, there was a practice session comprising six trials similar to experimental trials. Practice sessions were preceded by a familiarization phase, where participants were introduced to the shape, color, and color names of the stimuli (red, blue, orange, yellow, brown, green, gray, purple, and black disks presented against a light-gray background). When analyzing the data, we included participants whose accuracy scores surpassed 90% and who did not incorrectly match the colors displayed onscreen with the colors mentioned in connective expressions for most trials in any experimental condition. Based on these criteria, we excluded responses from four volunteers. Data from six more volunteers were incomplete, as they were not tested on all three experiments. Statistical analyses of response times were performed for all correct ‘yes’ responses within two standard deviations from the individual means.

## Experiment 1

The first study investigated the preference for either a split or a fused representation of connective constituents, starting from reports in earlier studies (Dumitru, 2014; Dumitru and Joergensen, 2016) that visual stimuli displayed together onscreen, either simultaneously or in sequential fashion, are compatible with a fused representation of connective constituents into a single Gestalt, whereas visual stimuli displayed alternatively such that only one of them is visible onscreen at any given time, are compatible with a split representation of connective constituents into different Gestalts. We started from the assumption that accuracy scores and/or processing times of visual stimuli provide information about the properties of language simulations that describe them. In particular, we expected faster processing for visuals displays when they matched language simulations than when they did not. Further, we expected Gestalt-based properties of visual stimuli to provide information on the Gestalt-based properties of language simulations (e.g., whether ‘orange’ and ‘blue’ in ‘There is orange AND there is blue’ form one or two Gestalts).

For the present work, we predicted faster responses to sequentially presented stimuli for connectives whose simulations comprise one Gestalt, given that both stimuli remain onscreen, thus building a single perceptual unit. We also predicted faster responses to alternatively presented stimuli for connectives whose simulations comprise two Gestalts, given that each stimulus can be assigned to one of the two language Gestalts, which are better perceived when presented separately, rather than together. The study also investigated the preference for either a left-to-right or a right-to-left processing direction of connective constituents, starting from the assumption that the attention flow over connective expressions might unfold either from the first to the second constituent mentioned, or from the second to the first constituent mentioned. Since participants were all native speakers of English, we expected them to first process the stimulus to the left, and only afterwards the stimulus to the right, hence we predicted faster responses when stimuli unfolded sequentially from left to right in connective expressions for which attention proceeds from the first to the second constituent, and faster responses when stimuli unfolded sequentially from right to left in connective expressions for which attention proceeds from the second to the first constituent.

### Method

#### Participants

A total of 27 volunteering students participated in the AND, OR, and BUT connective conditions, 36 participated in the IF and ALTHOUGH connective conditions, and 32 participated in the BECAUSE and THEREFORE connective conditions. They were all native speakers of English and had (corrected-to-) normal vision.

#### Design

The experiment followed a 2 (Dynamics: alternative vs. sequential) × 2 (Direction: right-to-left vs. left-to-right) full factorial design.

#### Stimuli and Procedure

**FIGURE 1:**
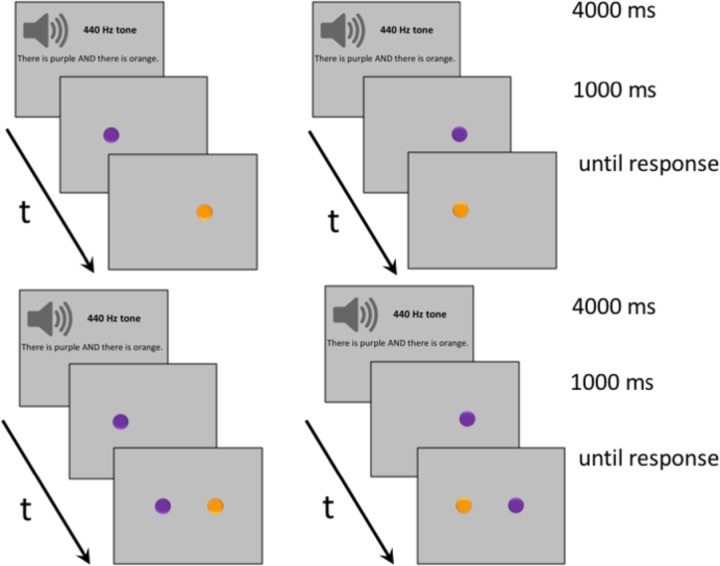
Examples of typical trials in Experiment 1. **(Upper)** Trials where participants listened to sentences containing one of the connectives AND, OR, BUT, IF, ALTHOUGH, BECAUSE, and THEREFORE, and then viewed visual stimuli displayed alternatively from right to left or from left to right. **(Lower)** Trials where participants listened to connective sentences and then viewed visual stimuli displayed sequentially.

Visual stimuli consisted of 280 dynamic horizontal displays of two differently colored disks. The displays were distributed over 7 connective conditions (AND, OR, BUT, IF, ALTHOUGH, BECAUSE, and THEREFORE), each of them covering four types, as follows. For two display types, stimuli appeared alternatively on the screen, either from left to right or from right to left. For the other two display types, stimuli appeared sequentially on the screen, again either from left to right or from right to left, as seen in **Figure 1**. When stimuli appeared alternatively, a single disk was visible onscreen at any given moment. When stimuli appeared sequentially, the first disk would be visible onscreen, followed by the second disk, such that both disks would remain visible. Auditory stimuli consisted of an equal number of basic sentences recorded by a male native speaker of English. Each sentence mentioned two different colors linked by one of the seven connectives (e.g., ‘There is purple AND there is orange,’ ‘There is purple OR there is orange,’ ‘There is purple BUT there is orange,’ ‘There is purple IF there is orange,’ ‘There is purple ALTHOUGH there is orange,’ ‘There is purple BECAUSE there is orange,’ ‘There is purple THEREFORE there is orange’). In half of the trials, both stimuli matched the colors mentioned in the connective sentence; in the other half, both stimuli mismatched the colors mentioned.

On a typical trial, subjects started by fixating a blank screen for 500 ms before hearing a 440 Hz tone for 400 ms, followed by a 600 ms pause, and by a connective sentence. The sentence always lasted 3000 ms, including a variable pause of a couple of hundred ms at the end. Afterwards, participants viewed two disks appearing onscreen either alternatively (a disk first appeared to the right, then it disappeared and another disk appeared to the left, or a disk first appeared to the left, then it disappeared and another disk appeared to the right) or sequentially (one disk appeared to the right, and another disk was added to the left, or one disk appeared to the left, and another disk was added to the right). The second disk always became visible 500 ms after the first disk appeared. The final display remained onscreen until response by button press. Participants selected the right button of a response box to signal a match between the colors of the disks and the colors mentioned in the connective sentence; they selected the left button to signal a mismatch (counterbalanced).

### Results

**FIGURE 2:**
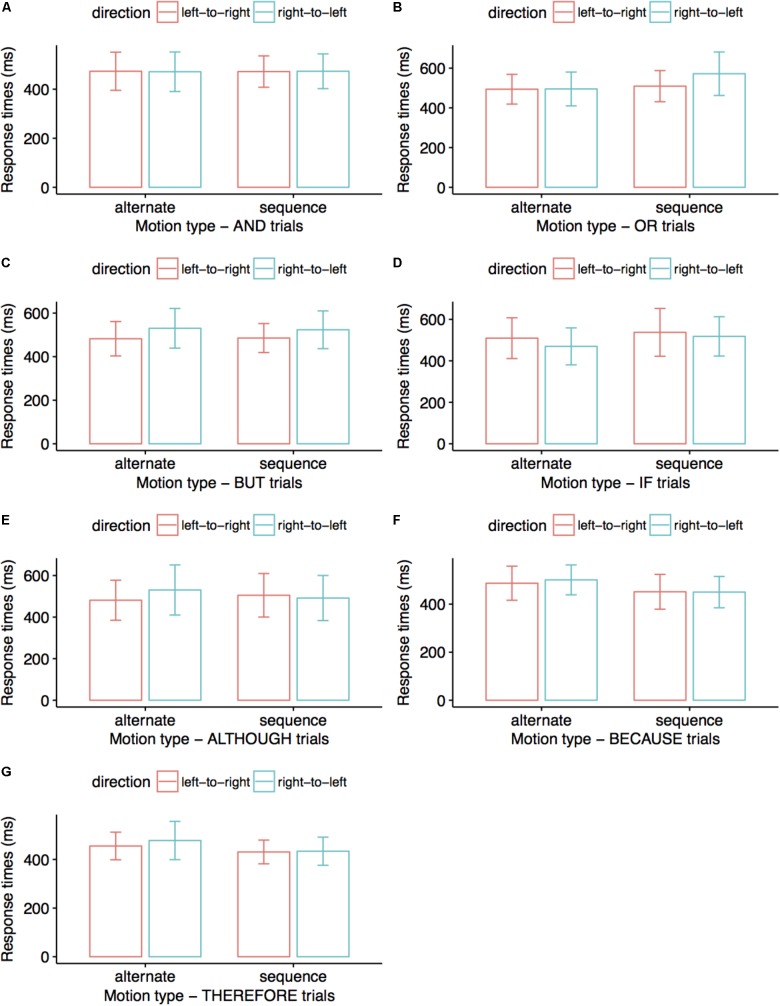
Average response times (ms) in Experiment 1. Participants matched the colors of visual stimuli to the colors mentioned in connective sentences. Error bars indicate 95% confidence intervals. Visual stimuli displayed alternatively were matched to OR sentences faster than visual stimuli displayed sequentially **(B)**; left-to-right displays were matched faster to BUT sentences than right-to-left displays **(C)**; alternative left-to-right displays were matched faster to ALTHOUGH sentences than alternative right-to-left displays **(E)**; sequential displays were matched faster to THEREFORE sentences than alternative displays **(G)**. No significant differences are presented in **(A)**, **(D)**, and **(F)** trials.

After removing all incorrect trials, we analyzed 96% of the data for AND, OR, BUT, and BECAUSE conditions, 97% of the data for the ALTHOUGH and THEREFORE conditions, and 98% of the data for the IF condition. We entered all response times in a 2 (Dynamics: alternative vs. sequential) × 2 (Direction: right-to-left vs. left-to-right) within-subjects ANOVA. **Figure 2** summarizes response times across conditions. For the connective OR, alternative displays were matched to spoken sentences faster than sequential displays, *F*(1,26) = 5.20, *p* = 0.031, η_p_^2^ = 0.167 (*M* = 494 vs. 541 ms). For the connective BUT, left-to-right displays were matched to spoken sentences faster than right-to-left displays, *F*(1,26) = 4.21, *p* = 0.050, η_p_^2^ = 0.139 (*M* = 484 vs. 527 ms). For the connective ALTHOUGH, we observed a marginally significant interaction between factors, *F*(1,35) = 3.44, *p* = 0.072, η_p_^2^ = 0.09 such that, for alternative trials, responses were faster in the left-to-right direction than in the right-to-left direction, *p* = 0.025 (*M* = 481 vs. 537 ms). For the connective THEREFORE, sequential displays were matched to spoken sentences faster than alternative displays (*M* = 442 vs. 474 ms), *F*(1,30) = 5.40, *p* = 0.027, **η_p_^2^** = 0.153.

There were no significant results for the connective conditions AND (*p*-values for the main factors ‘dynamics’ and ‘direction’ of 0.986 and 0.985, respectively, and *p* = 0.934 for their interaction), IF (*p*-values for the main factors ‘dynamics’ and ‘direction’ of 0.687 and 0.536, respectively, and *p* = 0.477 for their interaction), and BECAUSE (*p*-values for the main factors ‘dynamics’ and ‘direction’ of 0.303 and 0.882, respectively, and *p* = 0.978 for their interaction). There were further no significant effects for the main factor ‘direction’ or its interaction with the main factor ‘dynamics’ in OR trials (*p*-values of 0.241 and 0.166, respectively), for ‘dynamics’ or its interaction with ‘direction’ in BUT trials (*p*-values of 0.912 and 0.716, respectively), for the factors ‘dynamics’ or ‘direction’ taken separately in ALTHOUGH trials (*p*-values of 0.608 and 0.244, respectively), and for ‘direction’ or its interaction with ‘dynamics’ in THEREFORE trials (*p*-values of 0.506 and 0.777, respectively).

### Discussion

The results suggest that the simulations evoked by the connectives OR and ALTHOUGH include two Gestalts. Specifically, the connective OR triggered faster responses for alternative compared to sequential displays, and the connective ALTHOUGH showed sensitivity to the direction in which stimuli were alternated, which we cautiously take as circumstantial evidence for an overall preference for alternative rather than for sequential visual displays. The results also suggest that the simulations evoked by the connectives BUT and THEREFORE include a single Gestalt. Specifically, the connective THEREFORE triggered faster responses to sequential displays than to alternative displays and we cautiously considered the directional preference for sequential displays in BUT trials as circumstantial evidence that the connective simulation favors alternative over sequential processing of visual stimuli. The results also highlight a preference for the left-to-right default processing direction and hence for the order first-then-second constituent for attention allocation in BUT and ALTHOUGH conditions. Interestingly, response times indicate no clear preference for one or two Gestalts or their processing direction in the AND condition, which is hardly surprising considering that, in previous studies (e.g., Dumitru, 2014; Dumitru and Joergensen, 2016), results for AND trials were always quantitatively weak, at least when compared to results obtained for OR trials.

## Experiments 2A And 2B

The second study investigated the preference for allocating equal or unequal attention to simulation constituents in terms of matching connective sentences to either equal size stimuli (all large vs. all small) or to unequal size stimuli (one of them large, and the other small). A preference for all-large over all-small visual stimuli or the other way around in Experiment 2A would indicate that attention allocation encoded in simulations by specific connectives includes information about equal emphasis or equal de-emphasis. Equal emphasis would suggest that the two components in connective expressions are pitted against each other, or that they are both very important for deriving the meaning of connective expressions. A preference for a specific size trend in Experiment 2B that is, for either increasing or decreasing stimuli size, would indicate that the meaning of the connective contains instructions for attention to preferentially target one of the two constituents in connective expressions (i.e., either the second or the first), which is more important than the other, or which emphatically marks a simulation boundary that is, represents a cue for the point where the processing of connective expressions comes to an end.

When comparing participants’ performance across Experiments 2A and 2B, we assumed that equal-size stimuli are compatible with a fused representation of connective constituents into a single Gestalt, given that similar items are more readily thought of as originating from the same perceptual unit, whereas unequal-size stimuli would be compatible with a split representation of connective constituents into different Gestalts, given that dissimilar items are usually thought of as belonging to different perceptual units. Therefore, we predicted fast responses to equal-size stimuli accompanying connectives whose simulations comprise one Gestalt, as well as to unequal-size stimuli accompanying connectives whose simulations comprise two Gestalts.

### Method

#### Participants

There were 30 volunteers for the AND condition, 29 for the OR and BUT conditions, 28 for the IF and ALTHOUGH conditions, and 35 for the BECAUSE and THEREFORE conditions. As in the previous experiment, they were all native speakers of English and had (corrected-to-) normal vision.

#### Design

Experiment 2A contrasted stimuli size (small vs. large). Experiment 2B contrasted size trend (decreasing vs. increasing). We ran the experiments together but report the results separately, as factors in the two experiments were not crossed.

#### Stimuli and Procedure

Visual stimuli consisted of 280 dynamic displays of two concentric disks of different colors that appeared one after the other in the center of the screen, with the second disk overwriting the first. As before, displays were distributed over seven connective conditions (AND, OR, BUT, IF, ALTHOUGH, BECAUSE, and THEREFORE), each of them covering four display types, as follows. For two display types, disks were of equal size and were either small or large. For the other two display types, disk size either increased (the first disk was small and the second disk was large) or decreased (the first disk was large and the second disk was small). Auditory stimuli were the same as in Experiment 1 and consisted of an equal number of basic sentences, each of them mentioning two color names linked by one of the seven connectives (e.g., ‘There is purple AND there is orange’). In half of the trials, stimuli colors both matched the colors mentioned in connective sentences; in the other half, they both mismatched the colors mentioned.

**FIGURE 3:**
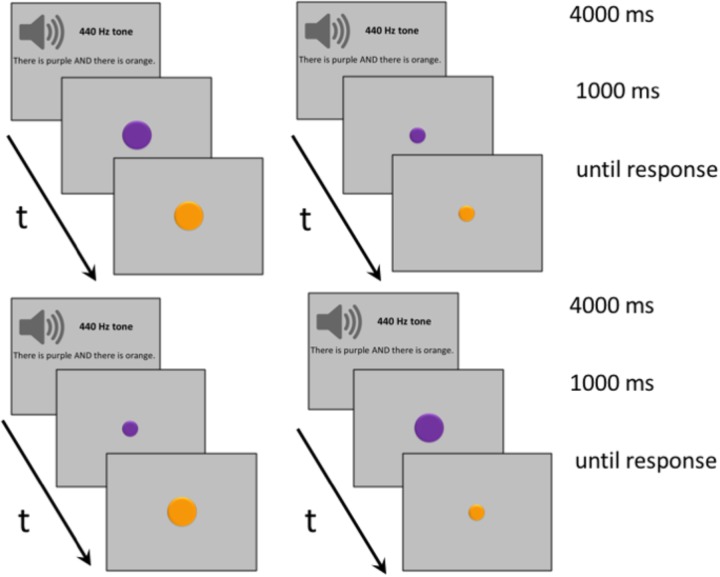
Examples of typical trials for Experiments 2A **(upper)** and 2B **(lower)**. **(Upper)** Trials where participants listened to sentences containing one of the connectives AND, OR, BUT, IF, ALTHOUGH, BECAUSE, and THEREFORE, and then viewed large or small stimuli pairs. **(Lower)** Trials where participants listened to connective sentences and then viewed visual stimuli either increasing or decreasing in size (i.e., the first stimulus presented was small and the second stimulus presented was large, or the other way around).

The procedure was similar to the one used in Experiment 1, with participants hearing a 440 Hz tone and a pause for a total of 1000 ms, and then a connective sentence for 3000 ms before viewing the two disks appear successively in the same location, as seen in **Figure 3**. The upper half of the figure shows disks of equal size; the lower half shows disks that differ in size, with either the first or the second disk being larger than the other. The delay between the first and the second display, which remained onscreen until response, was 500 ms. As in the previous experiment, responses to matching and mismatching trials were counterbalanced.

### Results

**FIGURE 4:**
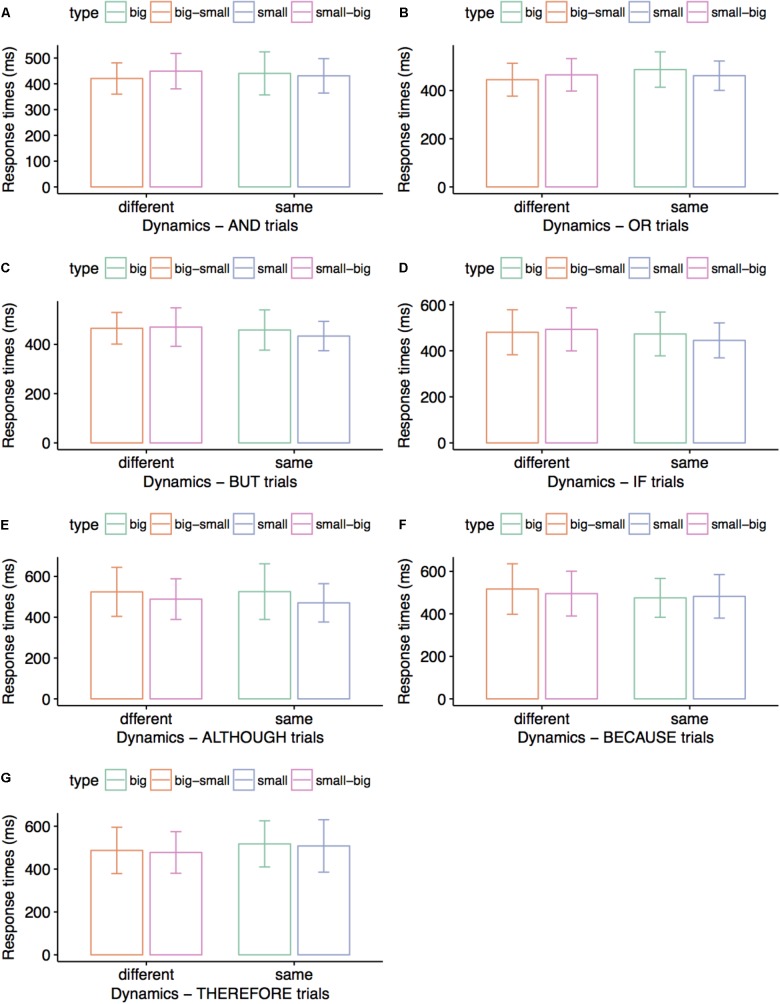
Average response times (ms) in Experiments 2A and 2B. Participants matched the colors of visual stimuli to the colors mentioned in connective sentences. Error bars indicate 95% confidence intervals. Small visual stimuli were matched to AND sentences faster than big visual stimuli **(A)**; different-size visual stimuli (big–small and small–big) were matched to OR sentences faster than either small visual stimuli or big visual stimuli **(B)**; big visual stimuli were matched to THEREFORE sentences faster than small visual stimuli **(G)**. No significant differences are presented in **(C–F)**.

**Figure 4** summarizes correct responses for matching connective sentences and visual displays across conditions. Correct responses averaged 98% for ALTHOUGH, BECAUSE, and THEREFORE conditions, and 99% for AND, OR, BUT, and IF conditions. Paired *t*-tests run for each experiment revealed marginally faster processing for small disks than for large disks in AND trials, *t*(29) = 2.03, *p* = 0.051 (*M* = 428 vs. 468 ms), and for large disks than for small disks in THEREFORE trials, *t*(34) = 2.11, *p* = 0.054 (*M* = 460 vs. 501 ms). Subsequently, we collapsed responses for equal–size stimuli in Experiment 2A and for different–size stimuli in Experiment 2B for each connective and found an overall processing advantage for different-size compared to equal-size disks in OR trials, *t*(29) = 2.09, *p* = 0.045 (*M* = 458 vs. 487 ms).

There were no significant results for the connectives BUT (*p*-values for comparing big and small disks, decreasing and increasing size, and same versus different-size disks of 0.493, 0.662, and 0.479, respectively), IF (*p*-values for comparing big and small disks, decreasing and increasing size, and same versus different-size disks of 0.582, 0.432, and 0.528, respectively), ALTHOUGH (*p*-values for comparing big and small disks, decreasing and increasing size, and same versus different-size disks of 0.132, 0.489, and 0.952, respectively), and BECAUSE (*p*-values for comparing big and small disks, decreasing and increasing size, and same versus different-size disks of 0.972, 0.182, and 0.105, respectively). We further obtained no significant results when comparing decreasing and increasing disk size, and same versus different-size disks in AND trials (*p*-values of 0.365 and 0.610, respectively), or when comparing decreasing and increasing disk size, and same versus different-size disks in THEREFORE trials (*p*-values of 0.665 and 0.193, respectively).

### Discussion

The results indicate that the simulations evoked by the connective OR include two Gestalts, as responses were faster for different-size than for same-size stimuli displays across trials. Results are less compelling in terms of indicating Gestalt numbers for simulations evoked by the connectives AND and THEREFORE, which may be considered to include one Gestalt based on faster responses in a subset of same-size stimuli trials. An interesting finding is the preference for all-small stimuli in AND conditions, and an opposite preference, for all-large stimuli, in THEREFORE conditions, which indicates that attention is targeting both constituents emphatically in simulations evoked by the latter but not by the former connective.

## Experiment 3

The third study investigated the preference for representing connective constituents in a specific orientation, namely horizontal, diagonal falling, diagonal rising, or vertical (i.e., two disks at the trigonometric angles of 180, 135, 45, or 90°), starting from the assumption that stimuli displayed vertically are compatible with a fused representation of connective constituents into a single Gestalt, whereas stimuli displayed horizontally are compatible with a split representation of connective constituents into different Gestalts. Even though both the up-and-down and the left-and-right orientations can map binary-valence concepts, we predicted that individuals would rely on embodied representations of two-Gestalt representations and re-enact, as it were, the gesture of assigning one Gestalt to left hand, and the other Gestalt to the right hand. We therefore predicted faster responses to visual stimuli whose orientation matched Gestalt arity in the simulations evoked by specific connectives. Further preference for one of the diagonal orientations over either the vertical or the horizontal would signal internal dynamics processes within a connective simulation or attention being allocated preferentially to one constituent of the simulation.

### Method

#### Participants

There were 32 volunteering students participating in the AND, OR, BUT conditions, 32 participating in the IF and ALTHOUGH conditions, and 29 participating in the BECAUSE and THEREFORE conditions. As in the previous experiments, volunteers were all native speakers of English and had (corrected-to-) normal vision.

#### Design

The experiment followed a 4 (Direction: horizontal vs. diagonal-falling vs. diagonal-rising vs. vertical) × 2 (Distance: proximal vs. distal) full factorial design for each of the seven connectives.

#### Stimuli and Procedure

**FIGURE 5:**
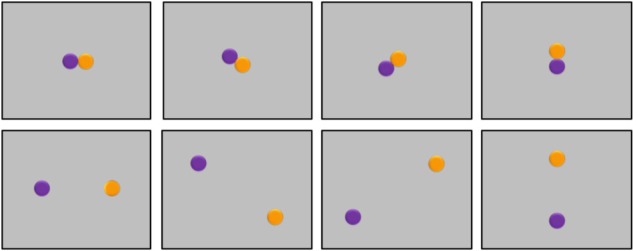
Examples of typical trials for Experiment 3. Participants listened to sentences containing one of the connectives AND, OR, BUT, IF, ALTHOUGH, BECAUSE, and THEREFORE, and then viewed stimuli pairs placed horizontally (180°), vertically (90°), diagonally upward (45°) and diagonally downward (135°), either close to each other **(upper)** or far away from each other **(lower)**.

Visual stimuli consisted of 448 stationary displays composed of two differently colored disks. Displays were distributed over seven connective conditions, each of them covering four display types. For each of them, stimuli were aligned according to one of four orientations, namely horizontal, diagonal-falling, diagonal-rising, and vertical. Half of the stimuli were displayed adjacent to each other (i.e., the proximal version), whereas the other half were displayed as far as possible from each other (i.e., the distal version), as seen in **Figure 5**, upper and lower quadrants, respectively. As in the previous experiments, participants were presented with an equal number of basic sentences mentioning two different colors, and one of the connectives AND, OR, BUT, IF, ALTHOUGH, BECAUSE, and THEREFORE (e.g., ‘There is purple AND there is orange’). In half of the trials, stimuli colors matched the colors mentioned in the connective sentence, whereas in the other half, they mismatched them.

The procedure was similar to the one used in Experiment 1 and in Experiment 2, with participants hearing a 440 Hz tone, a pause, a connective sentence, and another pause for a combined duration of 4000 ms, before seeing two disks appearing simultaneously on the screen in one of four orientations – horizontal, diagonal falling, diagonal rising, or vertical (i.e., at a trigonometric angle of 180, 135, 45, or 90°), as seen in **Figure 5**. In each trial, the display remained onscreen until response by button press (counterbalanced), for indicating a match or a mismatch between the colors mentioned in the sentence and the colors of the two disks.

### Results

**FIGURE 6:**
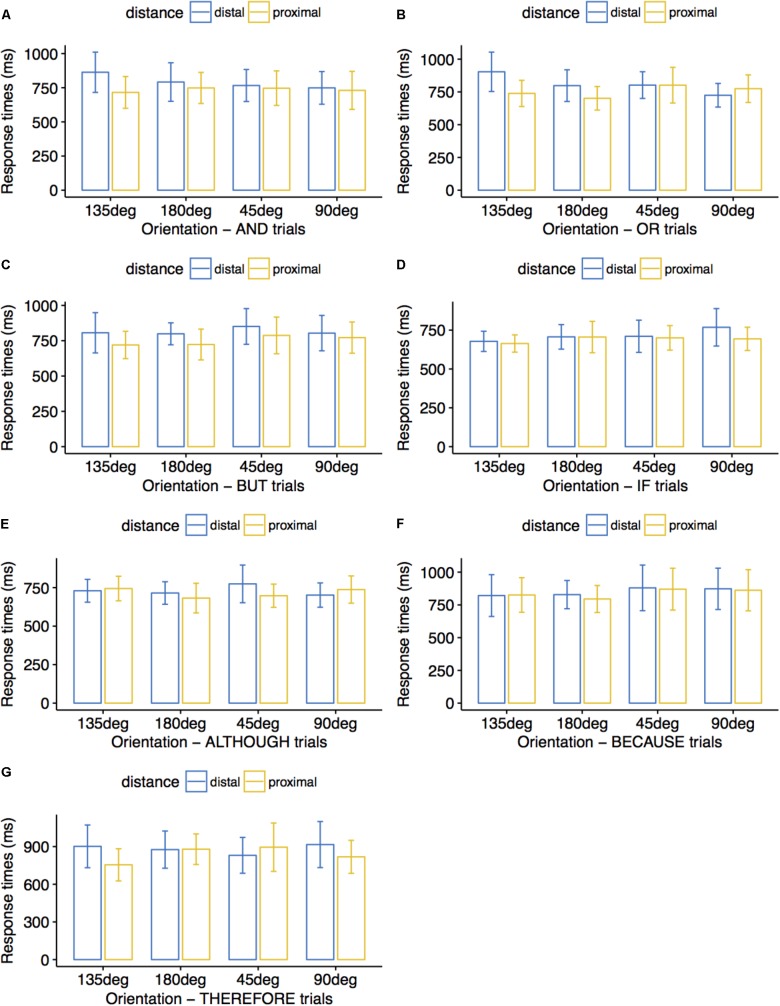
Average response times (ms) in Experiment 3. Participants matched the colors of visual stimuli to the colors mentioned in connective sentences. Error bars indicate 95% confidence intervals. Distal stimuli pairs forming a 45° diagonal were matched to AND sentences faster than distal stimuli pairs forming a 135° diagonal **(A)**; proximal stimuli pairs displayed at 180° were matched to OR sentences faster than proximal stimuli pairs displayed at 90° **(B)**; proximal stimuli pairs were matched to BUT sentences faster than distal stimuli pairs **(C)**; proximal stimuli pairs forming a 135° diagonal were matched to THEREFORE sentences faster than proximal stimuli pairs displayed at 180° and faster than proximal stimuli pairs forming a 45° diagonal **(G)**. No significant differences are presented in **(D–F)**.

**Figure 6** summarizes reaction time averages for correct trials across conditions for proximal and distal stimuli. Correct trials averaged 95% for the THEREFORE condition, 97% for the OR and the BECAUSE conditions, 98% for the BUT condition, and 99% for the AND, IF, and ALTHOUGH conditions. Within-subjects ANOVAs (4 directions × 2 proximity levels) run for each connective revealed overall faster processing for proximal compared to distal stimuli for AND trials, *F*(3,29) = 13.27, *p* = 0.001, η_p_^2^ = 0.300 (*M* = 735 vs. 793 ms), and a marginally significant interaction between direction and proximity, *F*(3,29) = 2.80, *p* = 0.057, η_p_^2^ = 0.225, with faster processing of distal stimuli displayed as a rising diagonal than as a falling diagonal, *p* = 0.043 (*M* = 766 vs. 863 ms) and of distal stimuli displayed as a vertical than as a falling diagonal, *p* = 0.046 (*M* = 748 vs. 863 ms). For OR trials, we obtained the same preference for proximal compared to distal stimuli, *F*(3,29) = 5.21, *p* = 0.029, η_p_^2^ = 0.144 (*M* = 754 vs. 807 ms), and an interaction between direction and proximity, *F*(3,29) = 9.77, *p* < 0.001, η_p_^2^ = 0.503, with faster processing of proximal stimuli displayed horizontally than vertically, *p* = 0.042 (*M* = 701 vs. 775 ms), as well as of distal stimuli displayed horizontally rather than falling, *p* = 0.035 (*M* = 798 vs. 903 ms), rising rather than falling, *p* = 0.031 (*M* = 802 vs. 903 ms), and vertically rather than falling, *p* = 0.001 (*M* = 724 vs. 903 ms). For BUT trials, we only observed an overall preference for proximal stimuli rather than for distal stimuli, *F*(3,29) = 9.41, *p* = 0.004, η_p_^2^ = 0.233 (*M* = 751 vs. 815 ms). For THEREFORE trials, we observed an interaction between direction and proximity, *F*(3,26) = 3.00, *p* = 0.049, η_p_^2^ = 0.257, with faster processing of proximal stimuli displayed as a falling diagonal than horizontally, *p* = 0.054 (*M* = 755 vs. 879 ms) or as a rising diagonal, *p* = 0.027 (*M* = 755 vs. 895 ms).

There were no significant results for the remaining connective conditions IF (*p*-values for the main factors ‘orientation’ and ‘distance’ as well as for their interaction were 0.261, 0.175, and 0.525, respectively), ALTHOUGH (*p*-values for the main factors ‘orientation’ and ‘distance’ as well as for their interaction were 0.366, 0.461, and 0.320, respectively), and BECAUSE (*p*-values for the main factors ‘orientation,’ ‘distance,’ and for their interaction were 0.303, 0.535, and 0.948, respectively). There were further no significant results for the main factor ‘orientation’ in AND trials (*p* = 0.603), for ‘orientation’ and its interaction with ‘distance’ in BUT trials (*p*-values of 0.230 and 0.714, respectively), and for ‘orientation’ in THEREFORE trials (*p* = 0.556).

### Discussion

The results indicate that simulations evoked by AND include one Gestalt, as responses were faster for vertical or diagonal-rising trials compared to horizontal or diagonal-falling trials, and that simulations evoked by OR include two Gestalts, as responses were faster for horizontal trials compared to vertical or diagonal-rising trials. Interestingly, there was an overall preference for proximal over distal stimuli even for the connective OR, whose simulation had been clearly shown to comprise two Gestalt (Dumitru, 2014; Dumitru and Joergensen, 2016). We propose to interpret these findings as an overall preference for processing visual displays where stimuli are not extremely distant from each other. As for the preference for stimuli forming a falling diagonal in the THEREFORE condition, it might suggest a boundary in the attention flow from the first to the second connective, corresponding to a final boundary in the simulation construction process, and thereby a definite boundary of the two-Gestalt simulation.

## General Discussion

The results of three studies investigating frequently occurring logical connectives (AND, OR, BUT, IF, ALTHOUGH, BECAUSE, and THEREFORE) provide evidence that rich embodied representations of logical connectives are instantly evoked when individuals listen to connective sentences without attempting to apply reasoning rules to determine whether there is a match or a mismatch between utterances and visual stimuli. In previous studies illustrating the Gestalt-based approach to language simulations (Dumitru et al., 2013; Dumitru, 2014; Dumitru and Joergensen, 2016), participants were asked to assess truth value satisfaction when matching visual displays to expressions containing logical connectives. In the studies reported here, participants merely listened to color words linked by one of the seven connectives, were not asked to retrieve the meaning of the connective, and the task itself did not require using the meaning of the connective when matching the two color-words to the visual stimuli presented onscreen.

Previous studies inspired by theories of embodied and grounded cognition provided evidence that lexical items such as ‘rose’ encode rich multimodal simulations of corresponding objects (Zwaan et al., 2002), and that language structure encodes attention patterns corresponding to gaze patterns over objects in visual scenes. Indeed, language and vision share cognitive resources (Richardson et al., 2003; Kaschak et al., 2005) and neuronal networks (e.g., Pulvermuller, 1999). For example, active and passive voice sentences (“The shark eats the fish” and “The fish is eaten by the shark,” respectively) evoke attention-related instructions for comprehenders to picture an event as being mainly about the agent or about the patient, whose perspective they should borrow when following with their gaze the objects presented in a visual scene (Tomlin, 1997). Experimental evidence further showed that inflected lexical items such as verb aspect encode event information. Thus, readers as well as comprehenders were more likely to associate perfective sentences (e.g., “The boy walked to the store”) but not imperfective sentences (e.g., “The boy was walking to the store”) with pictures illustrating completed events than with pictures illustrating ongoing events (Madden and Zwaan, 2003). By indicating whether comprehenders should represent events either as incomplete or as completed, verb aspect directs attention to the development or rather to the end result of a particular event.

In summary, the language system has been shown to encode information about specific attention patterns in terms of syntactic structures (e.g., active vs. passive voice), morphological categories (e.g., verbal aspect), or thematic roles (e.g., agent vs. patient). What we have shown here is that lexical items such as logical connectives also encode attention patterns to simulations evoked by the words they link together. The simulations evoked by logical connectives provide instructions relating to the simulations of the constituents they link together that is, information about navigating visual scenes (e.g., from left to right or from right to left), about grouping or not grouping objects together in a single Gestalt, and about the items within groups needing emphasis. By varying the direction, size, and orientation of visual stimuli, we replicated and extended previous findings that connectives such as AND and OR evoke one and two Gestalts, respectively (Dumitru et al., 2013; Dumitru, 2014; Dumitru and Joergensen, 2016). We determined that the connective OR evokes two Gestalts, for being compatible with visual displays where stimuli popup proceeds alternatively rather than sequentially, where stimuli size is different rather than equal, and where stimuli placement follows the horizontal orientation above all others. We also identified visual cues suggesting that the connective AND evokes a single Gestalt, for being compatible with visual displays where stimuli placement follows the vertical or diagonal-rising orientation. Furthermore, we report novel evidence on Gestalt number for the connectives BUT and THEREFORE, whose processing was sensitive to visual cues relating to stimuli dynamics specific to single-Gestalt simulations (i.e., sequential rather than alternative).

The variety of perceptual compatibility effects elicited across the connectives investigated suggests that each of them selects for specific simulation properties and perceptual cues, as predicted by theories of embodied cognition. We may surmise that identifying clear perceptual compatibility patterns for simulations evoked by connective expressions demonstrates the predictability of connective meaning in terms of attention patterns, stimuli range, and traces in working memory. Conversely, connective simulations for which no stable perceptual compatibility patterns can be identified, as we found to be the case for the connectives IF and BECAUSE, may be highly dynamic, task-specific, or completely unpredictable.

There are further characteristics of attention patterns deployed over constituent simulations that could not be readily interpreted as cues to Gestalt number. Specifically, the compatibility of AND and THEREFORE simulations with equal-size visual stimuli (all-small and all-large, respectively) reflects (lack of) overall emphasis; the compatibility of BUT and ALTHOUGH simulations with the default left-to-right processing direction reflects the importance of biological or cultural experience in information processing; the compatibility of THEREFORE simulation with a diagonal-falling orientation marks a boundary for the second constituent and a conclusion for attention allocation processes. These novel attention-based dimensions of connective simulations pave the way toward a more comprehensive conceptualization of language simulations as being highly dynamic constructs described in terms of Gestalt arity as well as in terms of Gestalt shape, size, direction, and boundaries, and for which biological and cultural background are relevant dimensions. The use of a matching task between the colors mentioned in connective sentences and the colors of binary stimuli displayed onscreen is particularly appropriate for covertly eliciting simulation properties for connectives that would otherwise be difficult to investigate in a reasoning task. For example, it would be hardly possible to design visual displays and instruct participants to apply specific logical rules for unambiguously matching these displays to the meaning of the connectives BUT or ALTHOUGH.

The results of our studies also contribute to the ongoing debate in the attention-related literature on whether visual Gestalt formation occurs pre-attentively or not. According to earlier views on visual information processing, Gestalt rules must apply before attention can select specific objects in a visual scene (e.g., Julesz, 1981; Pomerantz, 1981; Treisman, 1982; Duncan and Humphreys, 1989). Moreover, patients suffering from visual neglect were shown to be able to group information in the neglected field of view, further confirming the idea that at least some grouping abilities are pre-attentive (Driver and Halligan, 1991; Grabowecky et al., 1993). A competing stream of research, however, argues that attention must be an essential ingredient for organizing perceptual stimuli into groups. Thus, irrelevant (grouped) information presented in unattended areas (e.g., in the background or in the periphery) can modulate attention allocation patterns (Han et al., 2005) and object selection (Mack et al., 1992; Rock et al., 1992), at least when ensuing groups need to be maintained in working memory for further processing (Moore and Egeth, 1997). Previously, we reported that responses to visual displays organized as single Gestalts were faster than responses to visual displays organized as two Gestalts, both when they were described by AND expressions, whose simulation comprises one Gestalt, or by OR expressions, whose simulation comprises two Gestalts (Dumitru and Joergensen, 2016). These findings fall out of global precedence effects in visual processing (e.g., Navon, 1977) such that global configurations pre-attentively dominate local features in visual pattern perception. We ultimately established that AND and OR evoke, respectively, one and two language Gestalts based on significant interactions between factors (2 connectives × 2 visual display types).

In the current study, we analyzed results for each connective separately, and instead of presenting and removing two disks simultaneously or alternatively to suggest one and two Gestalts, respectively, we first presented one disk and subsequently added a second disk, thus building one Gestalt, or removed the first disk before presenting the second disk, thus building two Gestalts. In other words, we overrun global precedence effects by delaying the time necessary to construct a single Gestalt. We identified attention patterns deployed over connective simulations that were not essential for determining the number of visual Gestalts. For example, AND sentences were most rapidly matched to small disks of equal size or to disks displayed vertically. However, we also observed a preference for attending to stimuli on the left side first when building simulations corresponding to either one-Gestalt or two-Gestalt visual displays, which is a top-down attention effect that, unlike the global precedence effect, is culturally determined and relevant to both language and visual information processing. Language Gestalts encode attentional patterns affecting both the grouping of information in working memory and the characteristics of visual groups, including Gestalt shape, processing direction, and shape boundaries.

## Author Contributions

MD conceived and designed the studies. MD and GJ contributed to stimuli preparation. GJ collected the data. MD performed the statistical analysis and drafted the manuscript. All authors contributed to manuscript revision, read and approved the submitted version.

## Conflict of Interest Statement

The authors declare that the research was conducted in the absence of any commercial or financial relationships that could be construed as a potential conflict of interest.
